# Satisfaction of parents of schoolchildren with various aspects of the food management system at schools: Data from Russia

**DOI:** 10.1016/j.dib.2020.105725

**Published:** 2020-05-20

**Authors:** Dmitriy V. Adamchuk, Anna A. Arinushkina, Sergey S. Neustroev

**Affiliations:** Institute of Education Management of the Russian Academy of Education, Moscow, Russia

**Keywords:** Sociology of education, School nutrition, Epidemiology, Public health, School catering

## Abstract

The paper presents a rich collection of data obtained as a result of a sociological survey of 230,880 parents from 85 constituent entities of the Russian Federation. The survey was held in 2019 by the Institute of Education Management of the Russian Academy of Education in conjunction with the Ministry of Enlightenment of the Russian Federation as part of the state task No. 073-00089-19-01 on the topic “Optimization of the Hot Food System in Educational Organizations Implementing General Education Programs (Regional Aspects, Best Practices).” In particular, the paper provides data on the opinions of parents about the organization of school meals for their schoolchildren. A total of three large aspects are covered: (1) the demand for school cafeteria services and payment for meals; (2) satisfaction of parents with various aspects of catering in educational organizations; (3) catering control and information work.

Specifications table

**Table:** 

Subject	Education
Specific subject area	Sociology of Education
Type of data	Tables, Figures
How data were acquired	We conducted a sociological survey of 230,880 people using a detailed electronic questionnaire. Data processing was carried out using the Excel 2019 spreadsheet editor, as well as the IBM SPSS Statistics 19 and StatSoft Statistica 6.0 statistical software packages.
Data format	Raw and aggregated.
Parameters for data collection	The electronic questionnaire was built taking into account the following key indicators: (i) catering formats; (ii) satisfaction with the quality of food; satisfaction with the quality of service; (iii) frequency of providing poor-quality food; (iv) means of payment for meals; (v) the presence of a public commission for catering, awareness of its work; (vi) parents’ opinion on the need for changes in catering; (vii) informational work at the school on healthy nutrition.
Description of data collection	First, we designed a questionnaire to collect data on all indicators characterizing parents’ satisfaction with various aspects of the food management system at schools. Second, our questionnaires were sent via the Ministry of Enlightenment of the Russian Federation to all constituent entities of the Russian Federation. Regional ministers provided information to local schools. Parents were asked to fill out our questionnaire online. Third, all responses from parents were processed manually and converted into relevant datasets.
Data source location	85 constituent entities of the Russian Federation.
Data accessibility	The both raw and aggregated data for all 230,880 questionnaires from 85 constituent entities of Russia are in this data article as supplementary files. These data are fully financed and, consequently, fully possessed by the Ministry of Enlightenment of the Russian Federation. We collected and analyzed these data as part of the State Assignment No. 073-00089-19-01 “Optimization of the Hot Food System in Educational Organizations Implementing General Education Programs (Regional Aspects, Best Practices).”. In case of any queries about these data, one can contact directly Eteri V. Mindzaeva, Head (Manager) of the State Assignment. Email: project@iuorao.ru. Tel: +7 (495) 635 20-24. Address: Federal State Budgetary Institution “Institute of Education Management of the Russian Academy of Education”, 16 Zhukovskogo str., Moscow 105062 Russia.

## Value of the Data

• This is the most up-to-date and comprehensive dataset on the state of public opinion with respect to the whole range of issues existing in school food management systems on the territory of the entire Russian Federation.
• Both researchers and practitioners could benefit from this dataset by focusing on particular regions or specific issues within the food management systems.
• This dataset is of the highest value for (a) a continuous monitoring of the state of parents’ opinion on the issue; (b) any further interdisciplinary research in the fields of Sociology of Education, Public Health, or Education Management in the Russian Federation.

## 1 Data Description

This section of the paper fully describes the data obtained by us as a result of a sociological survey of 230,880 parents from 85 constituent entities of the Russian Federation. The survey was approved by the Human Research Ethics Committee of the Institute of Education Management of the Russian Academy of Education (Moscow, Russia).

The survey held in 2019 by the Federal State Budgetary Institution for Education Management of the Russian Academy of Education. A total of three large aspects are covered: (1) the demand for school cafeteria services and payment for meals; (2) satisfaction of parents with various aspects of catering in educational organizations; (3) catering control and information work. All the data are available in Appendixes A (questionnaire), B (aggregated data), and C (raw data).

Since the sociological survey of parents was conducted using the online survey tool “SurveyMonkey,” the raw data was initially presented in “.csv” and then was converted to “.sav.” The raw dataset contains all the respondents’ answers to the questionnaire. All code names begin with “QQn” and correspond to the questionnaire questions (No. 1-21), where “n” is the sequence number of the questionnaire. The raw dataset also has additional variables: (1) “QQ33_REG” reflects the respondent's affiliation to one of 85 constituent entities of the Russian Federation; (2) “QQ35_NP” captures the respondent's type of settlement; (3) “FED_OKR” is a transcoded “QQ33_REG” to reflect all federal districts of the Russian Federation (8 in total).

The rest of this paper presents the aggregated data only.

### 1.1 The demand for school cafeteria services and payment for meals

**Fig. 1:**
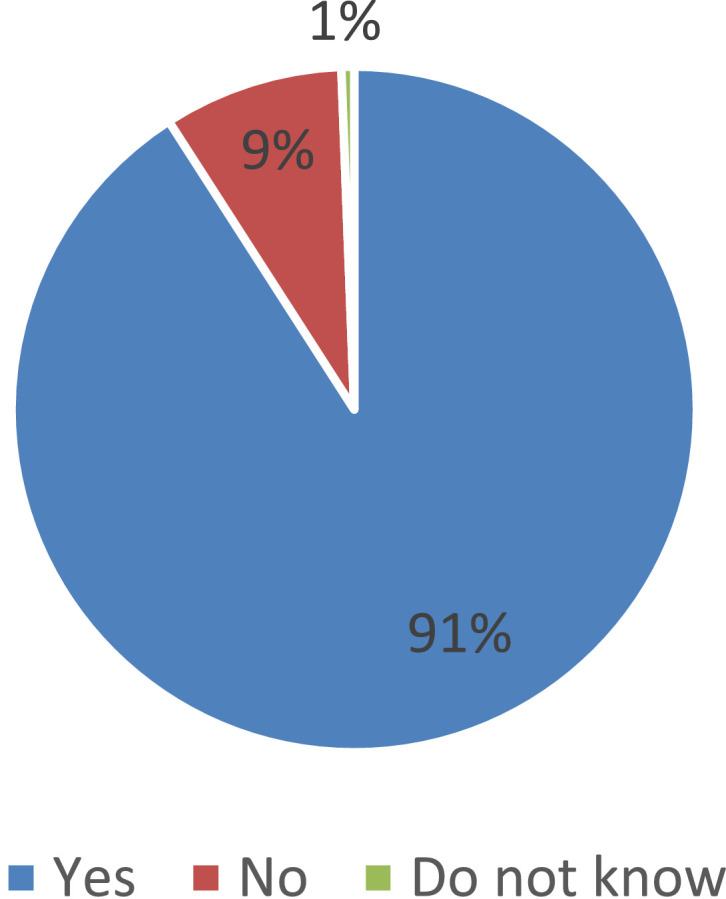
Parents’ answers to the question of whether the child uses the services of the school cafeteria or buffet (%).

**Fig. 2:**
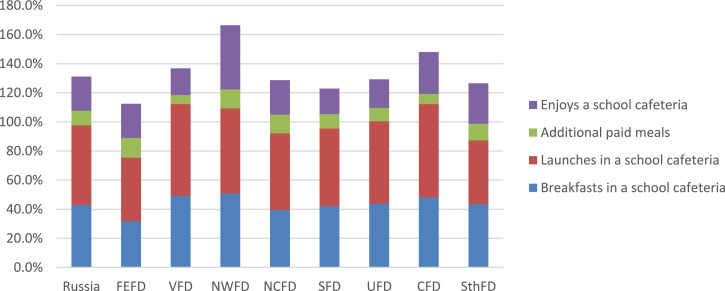
Parents’ answers about the main types of school canteen services that their child uses (%).

According to our sociological survey, 91% of parents said that their child use the services of a school cafeteria or buffet. According to the answers of parents, only 9% of students do not use the school cafeteria regularly (Fig. 1). At the same time, school lunches and breakfasts are the most demanded canteen services; these services are noted by 54.8% and 42.9% of parents on average in the Russian Federation. In fact, a quarter of parents (23.5%) report that their child uses buffet services. Moreover, the demand for buffet services for schoolchildren varies significantly in different federal districts of the Russian Federation (see Fig. 2).

**Fig. 3:**
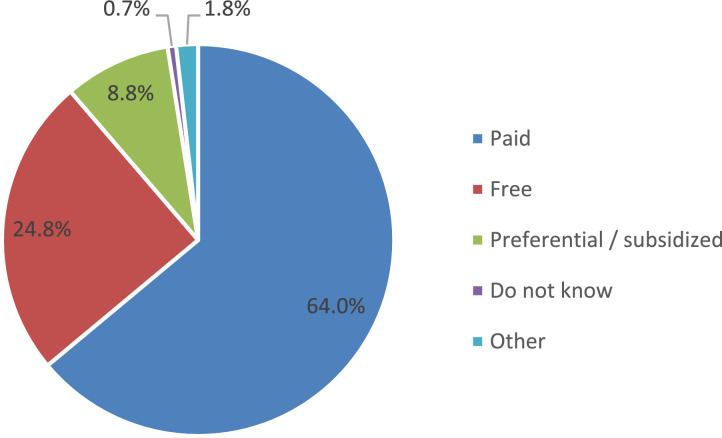
Parents' answers about school meals (%).

Almost two-thirds of parents (64%) report that food for their child is paid. One out of four respondents (24.8%) reports that their child eats at school for free. The presence of benefits when paying for food was noted by 8.8% of parents (Fig. 3).

**Fig. 4:**
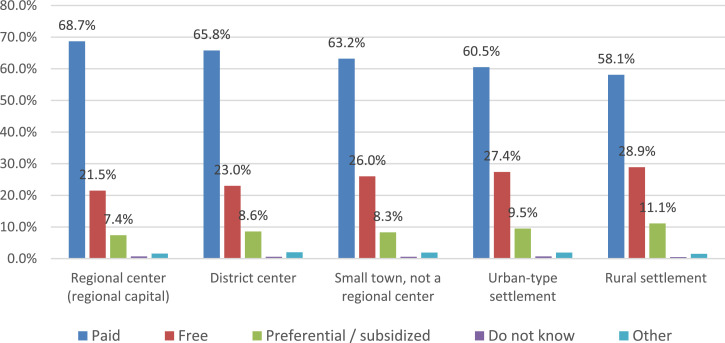
Settlement specificity of parents' answers about school meals (%).

**Fig. 5:**
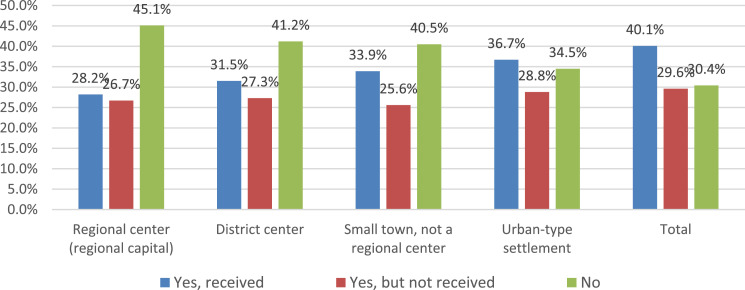
Settlement specificity in parents’ answers to the question “Do you think that your child should be provided with free food according to the income of your family?” (%)

Fundamental differences in the answers to the question of food payment are observed among respondents from various types of settlements (Fig. 4). The data presented indicate that an increasing share of parents indicates free school meals or the preferential nature of its payment with a decrease in the size (status) of the settlement. This trend undoubtedly indicates a high prevalence of social support measures among residents of small settlements. This fact is confirmed by the parents’ answers to the question of whether they believe that their child should be provided with free food as a measure of social support for the family based on her financial situation (Fig. 5).

**Fig. 6:**
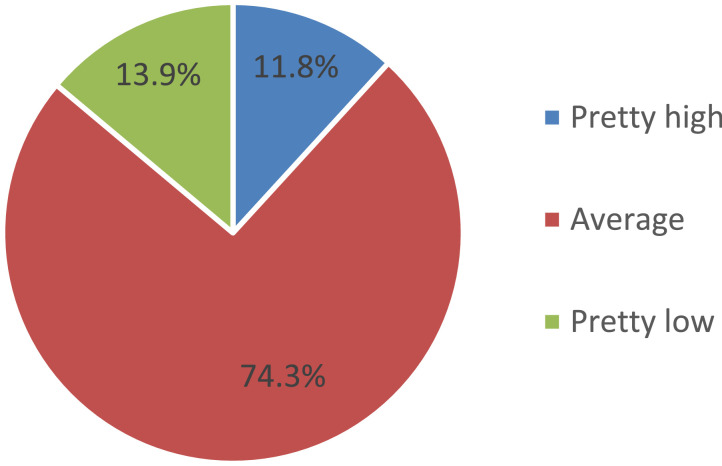
Evaluation of the level of prices for school cafeteria services by parents (%).

From the data shown in the figure, it can be seen that social support measures for school meals correspond to the expectations of respondents from various types of settlements to a rather high degree. So, the proportion of parents who believe that their child should receive food for free (but does not get it) varies slightly depending on the type of settlement. At the same time, in small towns there is a higher proportion of parents reporting that their child should receive free food based on the level of family income Fig. 6.

Assessing the price level for food in the school canteen, three quarters of parents (74.3%) indicated that it was “average,” and every seventh person being surveyed (13.9%) noted that the price level was “rather low.” Only 11.8% of parents believe that the price level in the school cafeteria is “very high.” At the same time, it is significant that 69.7% of parents (on average in the Russian Federation) express their willingness to pay a higher amount for the child's food while improving the quality of food (Fig. 6).

**Fig. 7:**
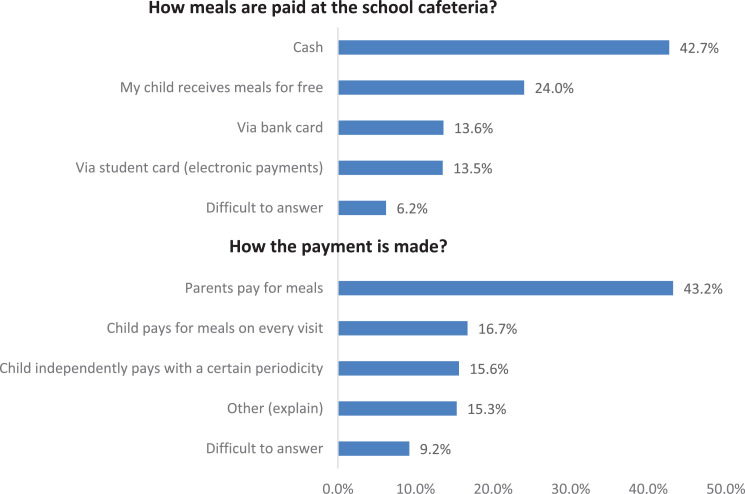
Parents’ answers about the organization of payments for food in the school cafeteria (average for the Russian Federation, %).

As for the payment mechanisms for the child's food in the school cafeteria, the most common payment option is cash payments (42.7%), while non-cash food payment schemes (“bank card,” “student card”) are noted by 27.1% of parents in total (Fig. 7).

Parents’ answers also indicate that payments for meals are usually carried out by the parents themselves (43.2%). In total, 32.3% of parents indicate that children pay for meals on their own (the child pays for meals at each visit to the dining room – 16.7%; with a certain frequency – 15.6%).

### 1.2 Satisfaction of parents with various aspects of catering in educational organizations

**Fig. 8:**
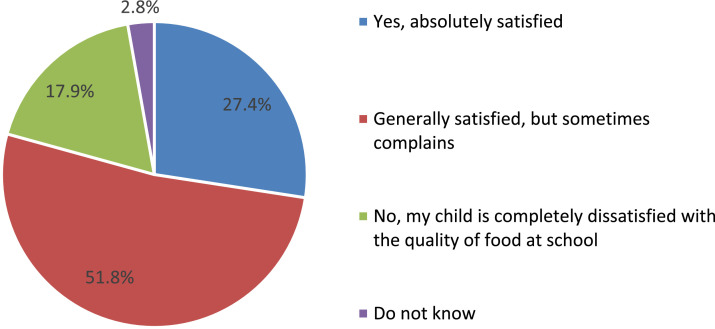
Parents’ answers about children's satisfaction with the quality of food in the school cafeteria (%).

**Fig. 9:**
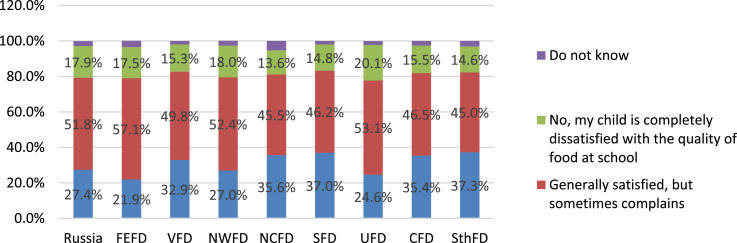
Parents' answers about their children's satisfaction with the quality of food in the school canteen in the federal districts of the Russian Federation (%).

The vast majority of parents (79.3%) note that their child is satisfied with the quality of food at school (Fig. 8). Moreover, comparisons of the answers of parents from different federal districts of the Russian Federation suggest that there are very significant differences in their estimates (Fig. 9).

The data allow us to compare most of the constituent entities of the Russian Federation (71 subjects) to obtain a conditional rating of satisfaction with school nutrition, which is based on such an indicator as the percentage of parents who note that their children are dissatisfied with school nutrition. Due to the statistical groundlessness of the data when analyzing materials on this issue, the answers of respondents from the following subjects of the Russian Federation were excluded from the sample: Kabardino-Balkarian Republic, Kamchatka krai, Kostroma region, Lipetsk region, Magadan region, Nenets Autonomous Okrug, Oryol region, Republic of Dagestan, Komi Republic, Republic of Tyva, Chechen Republic, Chukotka Autonomous Okrug, Yamalo-Nenets Autonomous Okrug, Yaroslavl region. For greater clarity, the distribution of the constituent entities of the Russian Federation by the proportion of parents indicating their child's dissatisfaction with food in the school cafeteria was divided into quartile groups (Table 1).

**Table 1:** The proportion of parents reporting that their child is dissatisfied with the quality of food at school in the subjects of the Russian Federation (%, quartile group).

Subject of the Russian Federation	% of parents who report that their child is dissatisfied with the quality of food at school	Quartile
Kemerovo region	5,1%	**Q1**
Ryazan region	5,9%	
Smolensk region	7,4%	
Republic of Ingushetia	7,6%	
Republic of Ingushetia	8,1%	
Kursk region	8,2%	
Orenburg region	8,6%	
Novgorod region	8,7%	
Jewish autonomous region	9,1%	
Republic of Mordovia	9,3%	
Kaluga region	9,5%	
Bryansk region	9,7%	
Altai krai	10,0%	
Vologodskaya region	10,1%	
Republic of Kalmykia	10,1%	
Vladimir region	10,3%	
Primorsky krai	10,3%	
Rostov region	10,4%	
Chuvash Republic	10,6%	**Q2**
Republic of Adygea	11,0%	
Republic of Sakha (Yakutia)	11,1%	
Belgorod region	11,2%	
Tomsk region	11,6%	
Karachay-Cherkess Republic	11,9%	
Kirov region	12,1%	
Irkutsk region	12,2%	
Transbaikal krai	12,5%	
Astrakhan region	12,7%	
Republic of Crimea	12,8%	
Volgograd region	13,2%	
Chelyabinsk region	13,5%	
Pskov region	14,1%	
Penza region	14,3%	
Republic of North Ossetia-Alania	14,4%	
Republic of Mari El	14,7%	
Perm krai	14,9%	
Tula region	15,0%	**Q3**
Udmurtia region	15,0%	
Amur region	15,2%	
Nizhny Novgorod region	15,4%	
Arkhangelsk region	15,9%	
Stavropol region	16,2%	
Ivanovo region	16,7%	
Novosibirsk region	16,9%	
Leningrad region	17,1%	
Murmansk region	17,1%	
Saratov region	17,3%	
Republic of Tatarstan	17,5%	
Tyumen region	17,6%	
Khanty-Mansiysk Autonomous Okrug - Ugra	17,8%	
Republic of Khakassia	18,0%	
Kaliningrad region	18,1%	
Krasnoyarsk region	18,3%	
Tambov region	18,3%	
Republic of Bashkortostan	18,5%	**Q4**
Moscow region	18,8%	
Samara region	19,7%	
Omsk region	20,1%	
Kurgan region	20,4%	
Sakhalin region	20,7%	
Ulyanovsk region	20,8%	
Republic of Buryatia	20,9%	
Sverdlovsk region	20,9%	
Altai Republic	21,0%	
St. Petersburg	22,0%	
Republic of Karelia	22,2%	
Krasnodar region	23,1%	
Voronexh region	24,3%	
Sevastopol	24,6%	
Khabarovsk region	28,9%	
Moscow	43,8%	

It should be noted that the data presented in the table reflect exclusively the subjective opinions of parents about the nutrition of children at school, since the level of satisfaction is directly related to the level of claims, which in turn directly depends on the socio-cultural and economic situation in the region. Thus, the given rating reflects the correspondence of the real quality of the organization of school meals to the expectations of the parents (legal representatives) of students. It should be borne in mind that the expectations that the parent community makes for catering at school can also vary significantly in different regions, since they are directly related to the specifics of a particular region (average income, socio-cultural factors, regional socio-economic characteristics).

**Fig. 10:**
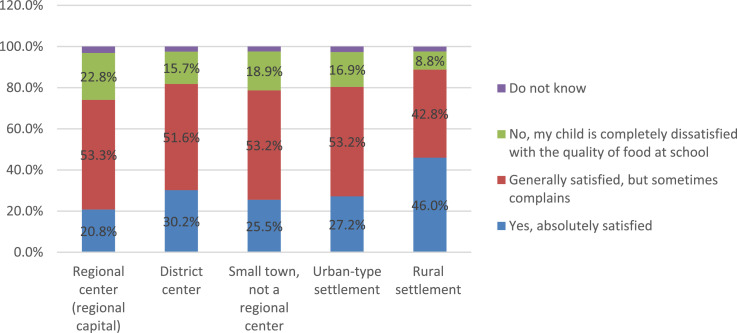
Settlement specificity in parents’ answers about children's satisfaction with the quality of food in the school cafeteria (%).

**Fig. 11:**
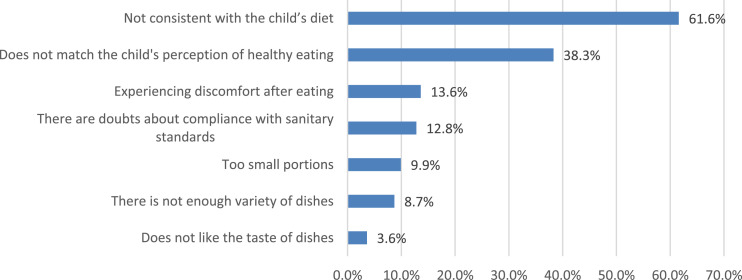
Parents’ answers about the main reasons for the child's dissatisfaction with the school cafeteria (%).

**Fig. 12:**
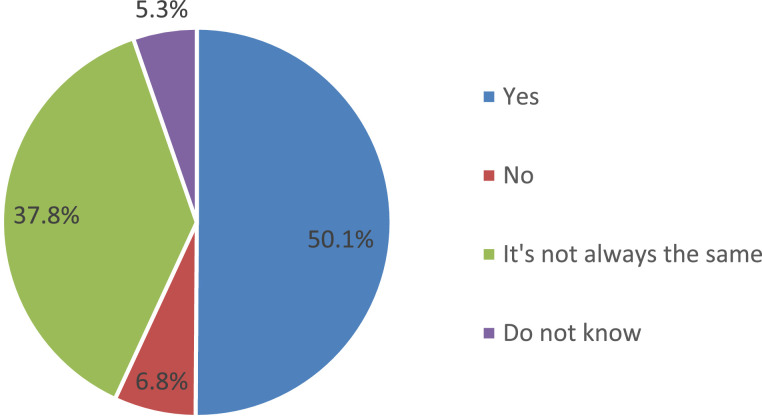
Parents' answers about children's satisfaction with the quality of service in the school cafeteria (%).

The socio-economic characteristics of satisfaction with food quality are especially evident when comparing the opinions of parents from different types of settlements (Fig. 10). Among the main reasons that cause dissatisfaction with the child's nutrition at school, parents note that “they do not like the taste of dishes” (61.6%), or the “lack of a sufficient variety of dishes” (38.3%) (Fig. 11). The remaining causes are noted by parents much less frequently. In addition to satisfaction directly with the quality of food, parents also answered about the organization of children's nutrition, namely about the quality of service in the school canteen (Fig. 12).

Fig. 11

Settlement specifics in the parents' responses are traced in relation to this aspect of school catering. So, while in large cities (regional centers) 43.0% of parents report child satisfaction with the service in the school canteen, the proportion of such answers is 68.2% in rural settlements.

**Fig. 13:**
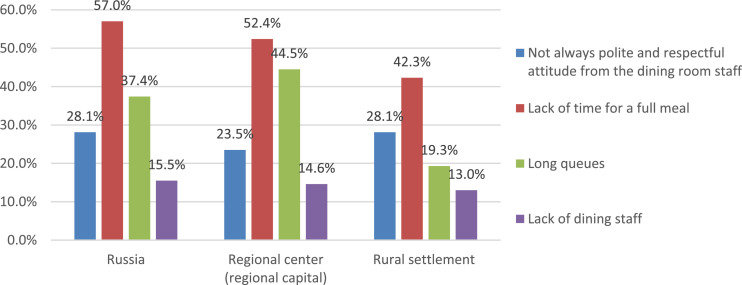
Parents’ answers about the main reasons for the dissatisfaction of the child with the service in the school cafeteria (%).

Of particular interest are the answers of parents about the main reasons for dissatisfaction with the service in the school canteen (Fig. 13). The main reasons for dissatisfaction with the work of the school cafeteria are issues related to the organization of meals. From the data shown in the figure, it can be seen that respondents from large cities are much more likely to report such reasons as “lack of time for eating” and the presence of “long lines.” In turn, respondents from rural settlements more often note problems associated with unpolite attitude of the canteen staff towards children.

**Fig. 14:**
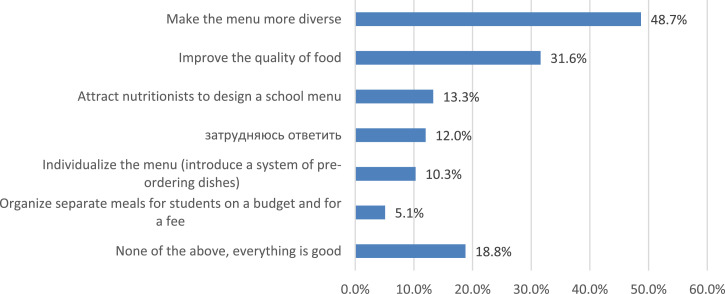
Opinions of parents about the necessary changes in the organization of school meals (%).

During the survey, parents also answered the question of what needs to be changed in the organization of food at school (Fig. 14). Almost every fifth parent (18.8%) does not see the need for any changes, noting that everything is already “good.” The most necessary changes from the point of view of parents concern the menu: “Make the menu more diverse” (48.7%); “Attract nutritionists to develop the menu” (13.3%); “Individualize the menu” (10.3%). In parallel with this, in fact a third of parents (31.6%) record the need for “improving the quality of food.”

**Fig. 15:**
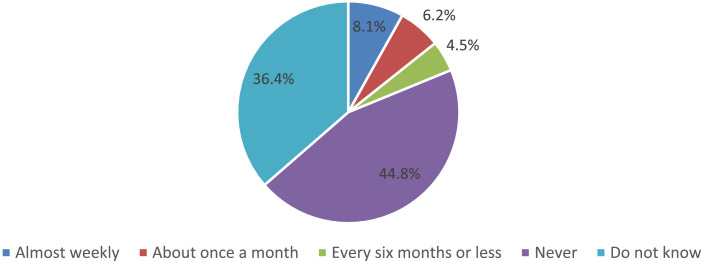
Parents’ answers to the question of how often their children are provided with poor-quality food in the school cafeteria (%).

During the survey, students’ parents were asked to answer the question of how often their children were offered poor-quality food in the school cafeteria (Fig. 15). The data presented in the figure indicate that a total of 18.8% of parents encounter this or that frequency on average in the Russian Federation with such situations. Moreover, more than a third of parents (36.4%) find it difficult to answer this question, because they are not aware of this. Thus suggests that their child simply does not express dissatisfaction with the quality of food in the school cafeteria; therefore, s/he did not encounter a high probability with similar cases.

**Fig. 16:**
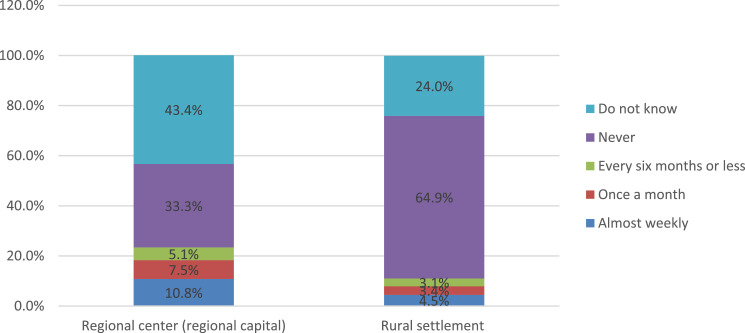
Parents’ answers to the question of how often their children are offered poor-quality food in the school canteen, depending on the type of settlement (%).

The settlement specificity in parents’ answers is also considered separately (Fig. 16). Among respondents from rural settlements, the proportion of those who are not aware of the work of the school canteen is almost half lower. Accordingly, there is a higher proportion of those who note that their child has never encountered such cases. It should be noted that respondents from large cities significantly more often indicate that their child has faced similar situations.

**Fig. 17:**
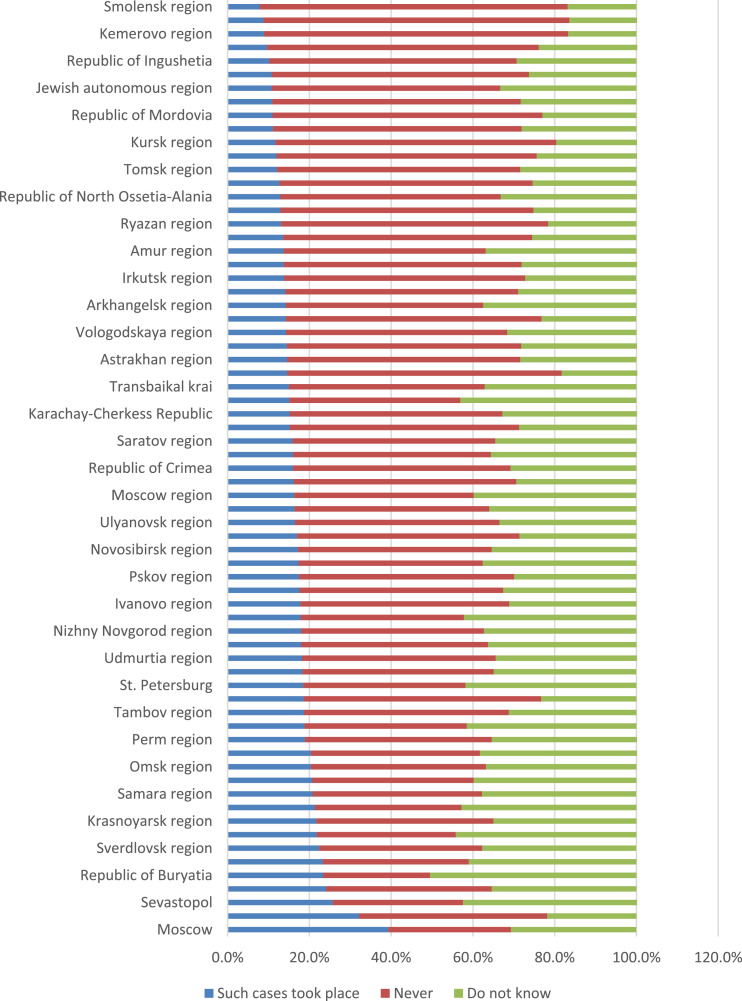
Parents’ answers to the question of how often their children are offered poor-quality food in the school cafeteria, depending on the subject of the Russian Federation (%).

In order to have an idea of the regional specifics in the parents’ answers to this question, we carried out an additional analysis, in which the data on cases of poor-quality food supply with one or another frequency were presented in total. In other words, the fact of the presence of such cases was recorded (Fig. 17). Least of all, our respondents encounter the fact that poor-quality food was served in school canteens in the Kaluga, Kemerovo, Orenburg, and Smolensk regions (less than 10% of respondents record such facts). Parents living in such regions as Moscow, Voronezh region, Sevastopol note such cases often (25% or more of respondents).

### 1.3 Catering control and information work

**Fig. 18:**
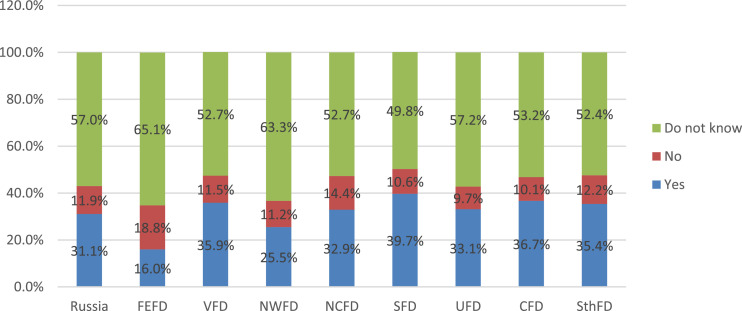
Parents’ answers to the question of the existence in their school of a public commission on organization and quality by federal districts (%).

Monitoring of the organization and quality of food by the parental community helps minimize the number of cases that cause discontent of parents and children with food at school [5,6]. In this regard, during the survey, parents were asked to answer the question about the existence of a public commission on the organization and quality of food in their school (Fig. 18).

The data presented in the figure indicate that more than half of the respondents (57%) find it difficult to answer this question on average in the Russian Federation since they do not have information about the work of the public nutrition commission at school. In parallel with this, almost a third (31.1%) indicates that such a commission exists in their school. The absence of such a commission is indicated by every eighth of the respondents (11.9%).

**Fig. 19:**
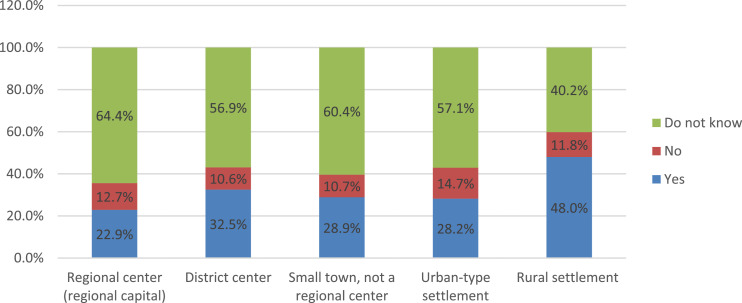
Parents’ answers to the question about the existence in their school of a public commission on organization and quality, depending on the type of settlement (%).

An analysis of the settlement specificity of the parents’ answers to this question indicates the presence of a pronounced trend: with a decrease in the size of the settlement, the parental community is becoming more active in fulfilling the functions of public control over the nutrition of schoolchildren (Fig. 19). In fact, one out of every two parents whose children are enrolled in rural schools, notes the existence of a public nutrition commission in an educational organization.

**Fig. 20:**
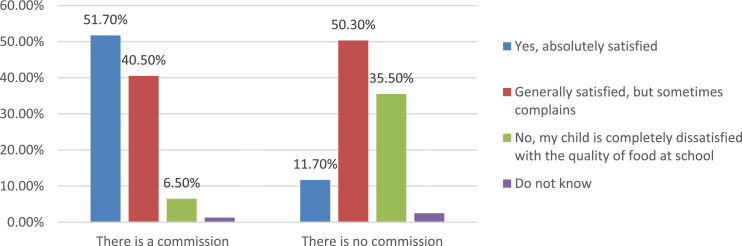
Comparison of parents’ answers about the level of child satisfaction with school meals, depending on the existence of a public commission on the organization and quality of nutrition at school (%).

**Fig. 21:**
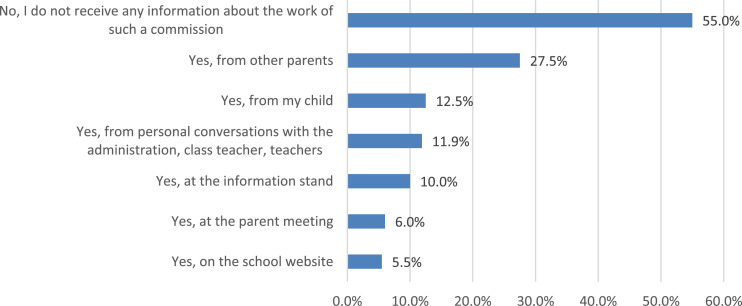
Parents’ answers to the question about sources of information about the work of the public commission (%).

The data of the survey allow a comparison of the parents’ answers about their child's satisfaction with the quality of nutrition in educational organizations in which the public nutrition commission exists and in organizations where it is absent (Fig. 20). More than half of parents do not receive any information about the work of the public nutrition commission at school (Fig. 21). The most popular sources of information about the work of the public commission are parent-teacher meetings, personal conversations with representatives of the educational organization, as well as educational organizations’ websites.

**Fig. 22:**
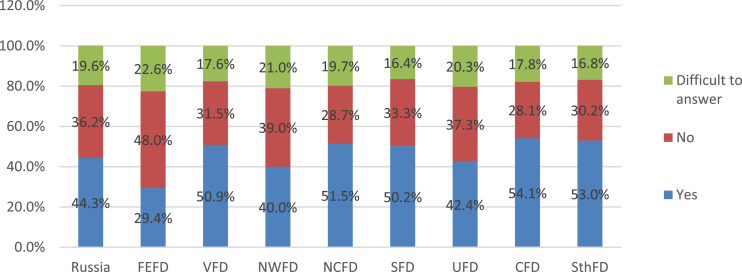
Parents’ answers about conducting conversations by school staff at parental meetings about a healthy lifestyle and the need for a balanced diet (%).

Answering the question “Would you like to take part in the work of the public commission to control the organization and quality of food at school?,” rhe vast majority of respondents (78.0%) answered negatively. Only one in five of the parents surveyed (22.0%) expresses a desire to take part in the work of such a commission. Despite this, informational work with parents about the nutrition of their child is carried out in educational institutions quite intensively (Fig. 22).

**Fig. 23:**
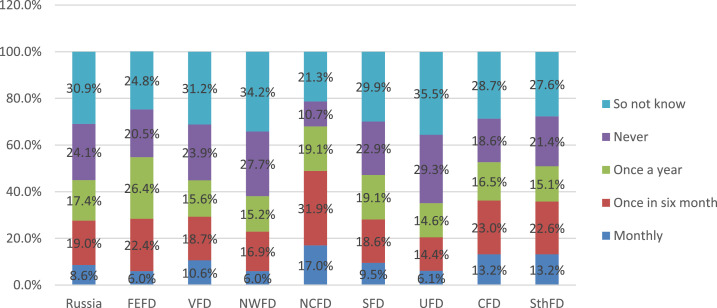
Parents’ answers about events (holidays, contests, fairs, etc.) related to cooking or healthy eating (%).

On average, almost half of the parents say that the school talks about the nutrition and healthy lifestyle of children. Another form of attracting the attention of the parental community and children to nutrition issues is various activities related to this issue. Parents’ answers to the question about the frequency of such events indicate that educational organizations are also very active in this area (Fig. 23).

## 2 Experimental Design, Materials, and Methods

Our sociological survey was conducted from April 1 to June 10, 2019, with the organizational support coming from the Ministry of Enlightenment of the Russian Federation. The research team sent out information letters to the regional education authorities of constituent entities of the Russian Federation asking them to invite parents of schoolchildren (or their legal representatives) to participate in the survey. In addition, official letters also contained the main parameters of the sample in the specific subject of the Russian Federation to which they were sent.

The main method of conducting a sociological survey among the parents was an electronic questionnaire (see Appendix A). Responses were received using the SurveyMonkey electronic service. They were downloaded in the “.csv” format and converted into the “.sav,” “.xlsx”, “.sta” formats by importing them into the corresponding software products. Data processing was carried out using the Excel 2019 spreadsheet editor, as well as the IBM SPSS Statistics 19 and StatSoft Statistica 6.0 statistical software packages.

If we talk about the content side of the sociological survey, the main indicators were the following aspects of catering in Russian schools: catering formats (the presence of a dining room, a buffet, vending machines); satisfaction with the quality of food (assortment, composition, taste, compliance with ideas about a healthy diet, etc.); satisfaction with the quality of service (courtesy of staff, presence / absence of conflicts, presence / absence of queues); frequency of providing poor-quality food; means of payment for meals (free / preferential / paid; cash / bank transfer; self-payment / payment by parents); the presence of a public commission for catering, awareness of its work; parents’ opinion on the need for changes in catering; informational work at the school on healthy nutrition (information stands, electronic resources, individual conversations with the health worker / class teacher).

Since obtaining representative data and organizing the collection of empirical material are fundamentally significant tasks in any research on nutrition [1,2], we paid special attention to building a sample at the stage of preparing a sociological survey. The study sampling plan was built on cluster-stratification principles. The development of the research sample was carried out in several stages. The first stage involved the implementation of the principle of clustering the general population. In total, 85 constituent entities of the Russian Federation comprising 8 federal districts were considered as clusters.

The second stage of sampling was associated with the application of the principle of stratification of the general population. The stratification was based on schoolchildren's age parameters, rather than the level of general education they receive (primary, basic, or high school). Thus, the number of students in a particular school and region was taken as the basis for calculating the optimal sample population. Based on this indicator, we calculated the optimal sample size with a confidence level of 95% and an error of 5% according to the Cochran formula [3].

The third stage in the development of the sample involved taking into account the settlement specifics of the region (urban / rural settlements) in the sample plan, for which the original sample was re-stratified by bringing the optimal sample to proportions of the urban and rural population based on the Rosstat data on estimating the resident population as of January 1, 2019 [4]. The total number of respondents from among parents and legal representatives of students who took part in a sociological survey was 230,880 people.

Variables characterizing the region, income level, and other characteristics that classify each respondent on various grounds were categorized based on their distribution. For example, a nine-point scale of self-assessment of the level of material security of the family was reduced to three categories, each of which accounted for 33.3% of the total variance of responses. The secondary variable “Federal District” was obtained by recoding the variable “Region” (Subject of the Russian Federation) to the value of the federal district corresponding to a particular subject. Other secondary variables were obtained in similar ways on the basis of mathematical and logical procedures.

Conjugation tables (see Appendix B) were built on the basis of two-dimensional distributions: a substantive question (row) and a sign (column). For example, a federal district or a type of settlement. Each cell in the contingency table (row and column intersection) reflects the number of respondents who have a certain attribute, indicates a corresponding answer option, as well as a percentage based on the total number of respondents related to the selected attribute.

## Declaration of Competing Interest

The authors declare that they have no known competing financial interests or personal relationships that could have appeared to influence the work reported in this paper.

## Appendix Supplementary materials

Image, application 1

Image, application 2

Image, application 3

Image, application 4
