# Supplementary material for: Satisfaction of parents of schoolchildren with various aspects of the food management system at schools: Data from Russia
Source: Data Brief. 2020 May 20;31:105725. doi: 10.1016/j.dib.2020.105725 (PMC7256467; doi:10.1016/j.dib.2020.105725)
Supplement: Supplementary file 1 [file mmc1.pdf]

Appendix A

Dmitriy V. Adamchuk, Anna A. Arinushkina, Sergey S. Neustroev
Institute of Education Management of the Russian Academy of Education, Moscow, Russia

SURVEY

Dear parent!

You are invited to participate in a survey. This survey will help to identify the opinions of parents of students about the organization of school meals.

We kindly ask you not to discuss your opinion with anyone when answering the questionnaire. This is important because it is your personal point of view that interests us.

Depending on the type of question, you can choose one or more statements as an answer, or enter your answer in the text field reserved for this.

If you missed a required question, the system will warn you about the need to give an appropriate answer.

Do not forget to click on the "End Survey" button at the end of the questionnaire. Otherwise, your results will not be saved!

When processing data, the research team guarantees you the complete anonymity of your answers.

If you have more than one school-age child, kindly answer about the nutrition of the child whose nutrition you are most informed about!

Thank you for participating in our survey. Your opinion is important to us!

1. What type of dining room is the school where your child is studying?

- ○ A full dining room
- ○ A pre-prepared dining room
- ○ A school base dining room
- ○ A sideboard
- ○ A school food factory
- ○ I find it difficult to choose the type (just some kind of school cafeteria)
- ○ Other form (please specify)_________________________

2. Does your child use a school cafeteria or buffet?

- ○ Yes
- ○ No
- ○ Do not know

3. What types of school canteen services does your child use?

- ☐ Breakfasts in a school cafeteria (first wave)
- ☐ Launches in a school cafeteria (first wave)
- ☐ Hot meals (second wave)
- ☐ Additional paid meals
- ☐ Enjoys a school cafeteria
- ☐ Products from vending machines

4. Meals in a school cafeteria for your child are:

- ○ Paid
- ○ Free
- ○ Preferential / subsidized
- ○ Do not know

5. Do you think that your child should be provided with free food based on the income of your family?

- ○ Yes, it is received
- ○ Yes, but my child does not receive
- ○ No

6. How are meals paid in the school cafeteria?

- ○ The child pays for meals on every visit to the canteen
- ○ The child independently gives money with a certain periodicity (once a week, a month, etc.)
- ○ Food for the child is paid by parents

7. How the payments are made:

- ○ Cash
- ○ Student card (electronic payments)
- ○ Bank card
- ○ My child receives meals for free
- ○ Difficult to answer

8. What is the price level for canteen services according to your opinion?

- ○ Pretty low
- ○ Average
- ○ Pretty high

9. Is your child satisfied with the quality of food?

- ○ Yes, absolutely satisfied
- ○ Generally satisfied but complains sometimes
- ○ No, my child is completely dissatisfied
- ○ Do not know

10. If your child is not satisfied with the quality of food at school, then what exactly is s/he complaining about?

- ☐ Experiencing discomfort after eating
- ☐ Does not match the child's perception of healthy eating
- ☐ There are doubts about compliance with sanitary standards
- ☐ Not consistent with the child's diet
- ☐ Does not like the taste of dishes
- ☐ Too small portions
- ☐ There is not enough variety of dishes
- ☐ Other reasons (please specify) ________________________

11. Is your child satisfied with the quality of service in his/her school canteen?

- ○ Yes
- ○ No
- ○ It is not always the same
- ○ Do not know

12. If your child is not satisfied with the quality of food at school, then what exactly is s/he complaining about?

- ☐ Not always polite and respectful attitude from the dining room staff
- ☐ Lack of time for a full meal
- ☐ Long queues

☐ Lack of dining staff

☐ Other (please specify) ______________________________

13. Do your children often get poor-quality food in the school canteen?

○ Almost weekly

○ About once a month

○ Every six month or less

○ Never

○ Do not know

14. What do you think should be changed in the organization of catering in your child's school?

☐ Make the menu more diverse

☐ Individualize the menu (introduce a system of pre-ordering dishes)

☐ Improve the quality of food

☐ Organize separate meals for students on a budget and for a fee

☐ Attract nutritionists to design a school menu

☐ Difficult to answer

☐ None of the above, everything is good

☐ Other (please specify) _________________

15. Are you ready to pay for a child's food (or increase the amount of payment) in case of fundamental improvements in the organization of school meals?

○ Yes, in case of reasonable prices

○ No, categorically not ready

16. Do health workers or other school staff talk at parental meetings about a healthy lifestyle and the need for a healthy diet?

○ Yes

○ No

○ Do not attend school meetings for parents

17. Does the school have a commission on the organization and quality of food?

○ Yes

○ No

○ Do not know

18. Do you receive information about the work of the public commission for monitoring the organization and quality of food at school?

☐ Yes, on the school website

☐ Yes, at meetings for parents

☐ Yes, at the information stand

☐ Yes, from personal conversations with the administration, class teacher, teachers

☐ Yes, from my child

☐ Yes, from other parents

☐ No, I do not receive information about the work of such a commission

19. Would you like to take part in the work of the public commission for monitoring the organization and quality of food at school?

○ Yes

○ No

20. Do you try to ensure that your nutrition at home is as healthy and complete as possible (enough vitamins, minerals, etc.)?

○ Yes, I pay a lot of attention

○ Yes, I try to make my nutrition complete if possible.

○ I do not pay much attention to this

21. Do you talk with your child about healthy eating habits (about wholesome and not wholesome foods, diet, etc.)?

○ Yes, regularly

○ Yes, from time to time

○ No, we do not discuss this topic

KINDLY PROVIDE INFORMATION ABOUT YOURSELF

22. How often does the school hold events (holidays, contests, fairs, etc.) related to cooking or healthy eating?

○ Monthly

○ Once in six month

○ Once a year

○ Never

○ Do not know

23. What region do you live in?

○ Republic of Adygea
○ Republic of Kalmykia
○ Republic of Kalmykia
○ Krasnodar krai
○ Astrakhan region

- ○ Volgograd region
- ○ Rostov region
- ○ Sevastol
- ○ Republic of Bashkortostan
- ○ Republic of Mari El
- ○ Republic of Mordovia
- ○ Republic of Tatarstan
- ○ Udmurtia Republic
- ○ Chuvash Republic
- ○ Perm krai
- ○ Kirov region
- ○ Nizhniy Novgorod region
- ○ Orenburg region
- ○ Penza region
- ○ Samara region
- ○ Saratov region
- ○ Ulyanovsk region
- ○ Republic of Altai
- ○ Republic of Buryatia
- ○ Republic of Tyva
- ○ Republic of Khakassia
- ○ Altai krai
- ○ Transbaikal krai
- ○ Krasnoyarsk region
- ○ Irkutsk region
- ○ Kemerovo region
- ○ Novosibirsk region
- ○ Omsk region
- ○ Tomsk region

24. Name the settlement in which you live: ____

25. Type of your settlement:

- ○ Regional center (regional capital)
- ○ District center
- ○ Small town, not a district center
- ○ Urban-type settlement
- ○ Rural settlement

26. Indicate the number (or official name) of the school in which your child is studying:

____

27. Indicate the type of educational institution in which your child is studying:

- ○ A principal comprehensive school
- ○ A middle comprehensive school
- ○ A school with in-depth study of specific subjects

- ○ A school with a higher status (lyceum, gymnasium, educational center)
- ○ Other (please specify): __________

28. What class is your child studying in?

- ○ 5
- ○ 6
- ○ 7
- ○ 8
- ○ 9
- ○ 10
- ○ 11

29. Age of your child (full years): ________________

30. Gender of your child:

- ○ Male
- ○ Female

31. Your educational level:

- ○ Secondary education (only school)
- ○ Vocational education (vocational school, college, technical school)
- ○ Higher education
- ○ Candidate or Doctor of Sciences

32. Rate the level of material security of your family on the proposed scale (1 - a very low level, 9 - a very high level):

1	2	3	4	5	6	7	8	9
○	○	○	○	○	○	○	○	○

Thank you for your responses!
